# Prognostic and clinicopathological significance of CD155 expression in cancer patients: a meta-analysis

**DOI:** 10.1186/s12957-022-02813-w

**Published:** 2022-10-29

**Authors:** Dan Zhang, Jingting Liu, Mengxia Zheng, Chunyan Meng, Jianhua Liao

**Affiliations:** 1 Department of Anesthesiology, Zhejiang Hospital, 12 Lingyin Road, Zhejiang, 310013 Hangzhou China; 2 Department of Health Management, Sir Run Run Shaw International Medical Centre, 9 Jingtan Road, Zhejiang, 310000 Hangzhou China; 3 Department of Surgery, Zhejiang Hospital, 12 Lingyin Road, Zhejiang, 310013 Hangzhou China

**Keywords:** CD155, Cancer, Prognosis, Biomarker, Meta-analysis

## Abstract

**Background:**

It has been previously reported that CD155 is often over-expressed in a variety of cancer types. In fact, it is known to be involved in cancer development, and its role in cancer has been widely established. However, clinical and mechanistic studies involving CD155 yielded conflicting results. Thus, the present study aimed to evaluate overall prognostic value of CD155 in cancer patients, using a comprehensive analysis.

**Methods:**

Online databases were searched, data was collected, and clinical value of CD155 was evaluated by combining hazard ratios (HRs) or odds ratios (ORs).

**Results:**

The present study involved meta-analysis of 26 previous studies that involved 4325 cancer patients. These studies were obtained from 25 research articles. The results of the study revealed that increased CD155 expression was significantly associated with reduced OS in patients with cancer as compared to low CD155 expression (pooled *HR* = 1.772, 95% *CI* = 1.441–2.178, *P* < 0.001). Furthermore, subgroup analysis demonstrated that the level of CD155 expression was significantly associated with OS in patients with digestive system cancer (pooled *HR* = 1.570, 95% *CI* = 1.120–2.201, *P* = 0.009), hepatobiliary pancreatic cancer (pooled *HR* = 1.677, 95% *CI* = 1.037–2.712, *P* = 0.035), digestive tract cancer (pooled *HR* = 1.512, 95% *CI* = 1.016–2.250, *P* = 0.042), breast cancer (pooled *HR* = 2.137, 95% *CI* = 1.448–3.154, *P* < 0.001), lung cancer (pooled *HR* = 1.706, 95% *CI* = 1.193–2.440, *P* = 0.003), head and neck cancer (pooled *HR* = 1.470, 95% *CI* = 1.160–1.862, *P* = 0.001). Additionally, a significant correlation was observed between enhanced CD155 expression and advanced tumor stage (pooled *OR* = 1.697, 95% *CI* = 1.217–2.366, *P* = 0.002), LN metastasis (pooled *OR* = 1.953, 95% *CI* = 1.253–3.046, *P* = 0.003), and distant metastasis (pooled *OR* = 2.253, 95% *CI* = 1.235–4.110, *P* = 0.008).

**Conclusion:**

Altogether, the results of the present study revealed that CD155 acted as an independent marker of prognosis in cancer patients, and it could provide a new and strong direction for cancer treatment.

**Supplementary Information:**

The online version contains supplementary material available at 10.1186/s12957-022-02813-w.

## Background

It has been previously reported that CD155 is often over-expressed in a variety of cancer types. In fact, it is known to be involved in cancer development, and its role in cancer has been widely established. However, clinical and mechanistic studies involving CD155 yielded conflicting results. Here, the present study aimed to comprehensively explore the relationship between CD155 expression and clinical characteristics and prognosis of cancer patients, thereby attempting to define the role of CD155 in various cancer types.

## Introduction

Cancer is the leading cause of death worldwide. In fact, it is a major public health concern [1]. It is known that cancer damages patient organ functions and extremely affects patients’ psychological and social relations, which further results in a significant deterioration of their quality of life. In order to reduce the impact of cancer on patients, a large number of studies have been conducted to explore the mechanism of occurrence and progression of cancer. In fact, great advances have been made in the prevention, surgery, chemotherapy, immunization, targeting, and other aspects of cancer. In the past few years, a number of different ways/strategies have been identified to ameliorate cancer burden. In particular, a number of markers have been identified that are either abnormally expressed in cancer or expressed only during cancer. These could partly explain cancer progression [2, 3]. However, tricky/complex nature of cancer has resulted in significant variations among races, regions, and organs, which in turn lead to conflicting results. Consequently, no significant improvement has been reported in terms of incidence and mortality associated with cancer, in the recent years [4]. Therefore, the present study aimed to identify a tumor marker that could guide clinicians in a relatively stable way and thus mitigate current dilemma to certain extent.

Certain molecules have been previously shown to be barely expressed in most of the normal tissues; however, these molecules exhibited up-regulated expression in a variety of human malignancies and played a vital role in cancer development. CD155 is one of these molecules, which is often over-expressed in cancer cells. It is known to be involved in various processes, such as cell adhesion, migration, proliferation, and tumor surveillance [5]. CD155 is also known as PVR, NECL-5, and TAGE-4, primarily owing to its different roles and attributions [6–8]. In particular, it has been previously shown that CD155 aggregates at the leading edge of migrating tumor cells and co-locates with actin and αvβ3 integrin to promote cancer cell migration [9]. Cancer cell dispersal was found to be enhanced by up-regulation of CD155 expression, which recruited Src homology region 2 domain-containing phosphatase and further potentiated focal adhesion kinase signaling [10]. Besides this, aberrant expression of CD155 could up-regulate cyclin D2 and shortened the G0/G1 phase. In comparison to this, down-regulated CD155 inhibited the proliferation of cancer cells and blocked the cell cycle at G2/M phase [11]. Additionally, previous studies reported that CD155 knockdown suppressed proliferation and promoted apoptosis via AKT/Bcl-2/Bax [12]. Similarly, differential expression of CD155 might regulate PDGF-mediated cell proliferation, VEGF expression, and intratumoral vascular density [13, 14]. However, accumulating evidences revealed that CD155 performed various other functions. In fact, it was reported that CD155 played a more complex role in tumor immunity and surveillance [15]. In particular, over-expression of CD155 in cancer is recognized by a group of receptors, including DNAM-1, TIGIT, and CD96, expressed on T and NK cells, which further transmit an alert signal to the immune system during malignant transformation. In brief, CD155 can be both immunostimulant if binding to DNAM-1 or immunosuppressant if binding to TIGIT or CD96. Notably, studies have shown that CD155 might interact with inhibitory receptors to attenuate DNAM-1-mediated signaling in advanced clinical stages, accompanied by upregulation of the inhibitory receptors TIGIT and CD96 and decreased expression of DNAM-1, which would further lead to inhibition of activation of NK and T cells and facilitate immune escape [16]. Although CD155 has the best affinity for TIGIT and tends to immunosuppress and reduce the activity of TIGIT expressing NK and T cells in the tumor microenvironment, the sensitivity of tumor cells to NK cell-mediated cytotoxicity is not only regulated by CD155/TIGIT [17–19]. In fact, it was reported that expression of CD155 mediated elimination of DNAM-1-dependent tumor cells by NK and CD8+ T cells. Additional evidences demonstrated that CD155 expression inhibited anti-tumor activity of tumor cells in DNAM-1-deficient mice, and over-expression of CD155 resulted in tumor rejection of/by NK cells, which was mediated by DNAM-1 [20, 21]. Moreover, CD155 expression, activated by DNA damage-response pathway, stimulated NK cell-mediated elimination of malignant plasma cells [22]. Remarkably, blockade of CD155 signaling has been previously shown to augment anti-tumor immunity [23]. The role of CD155 in cancer has been verified in vitro and in vivo experiments. Importantly, clinicopathological analysis concluded that CD155 expression was associated with prognosis of cancer patients [2, 3]. These findings highlighted that CD155 played a physiological role as a cancer-associated molecule. Altogether, CD155 played a critical in tumor progression; however, certain results were contradictory. A large number of studies have previously explored the clinical value of CD155; however, no previous study comprehensively analyzed CD155 expression and function in cancer patients.

The present study aimed to comprehensively explore the relationship between CD155 expression and clinical characteristics and prognosis of cancer patients. The study was based on comprehensive search of relevant literature. In particular, the study attempted to define the role of CD155 in various cancer types.

## Materials and methods

### Search strategy and study selection

We conducted systematic retrieval through PubMed, PMC, Web of Science, and other network databases until May 2022 and used the following retrieval formula: “CD155” AND “cancer OR tumor OR neoplasm OR carcinoma” AND “prognosis OR Prognostic OR survival OR outcome,” with the retrieval formula adjusted according to the format of different databases in the retrieval process. Meanwhile, other aliases of CD155 such as PVR, NECL-5, and TAGE-4 were also substituted in the retrieval formula and retrieved them one by one. We also attempted to retrieve the relevant researches from the references as much as possible. The retrieval process was independently perfected by two researchers, and the possible contradictions were resolved by a third researcher. Details of the protocol for this systematic review were registered on INPLASY (INPLASY202290087) and are available in full on the inplasy.com (10.37766/inplasy2022.9.0087). Indeed, the meta-analysis of this study was consistent with the reporting checklist as a part of the Preferred Reporting Items for Systematic Reviews and Meta-Analyses statement [24].

### Inclusion and exclusion criteria

The pre-established inclusion criteria were as follows: (1) all subjects were cancer patients who received standard treatment; (2) the expression of CD155 in the cancer patients was well-examined, and all patients were assigned into two groups based on the expression; (3) survival analysis was performed based on these two groups and provided sufficient data to estimate the risk ratio (HR) and 95% confidence interval (CI) for overall survival (OS); and (4) scientific and reasonable research. Case reports, reviews, abstracts, letters, bioinformatic analysis, TCGA analysis, and articles that did not meet the inclusion criteria were excluded from analyses.

### Data extraction and quality assessment

In the included literature, we collected the study data including authors, study region, sample size, cutoff scores, cancer type, and HR estimation, as well as the clinical data including age, gender, TNM stage, lymph node (LN) metastasis, distant metastasis, tumor size, and tumor grade. All data extraction was performed independently by two researchers and verified and aggregated by a third researcher. The Newcastle-Ottawa quality assessment scale was applied to assess the quality of the included studies in this study [25]. As following categories: selection, comparability, and exposure. Briefly, based on the sum of three categories, we considered studies as high quality if the score > 6.

### Statistical analyses

All statistical analyses were performed using the STATA 14.0 software, and two-sided *P* < 0.05 indicated statistical significance. The prognostic value of the included studies was assessed by HR. The collected HR and their corresponding 95% CI were integrated and pooled in SPSS software with established codes to estimate the association between CD155 expression and OS in cancer patients. Similarly, we evaluated the correlation between the CD155 expression and the clinical characteristics by pooling odds ratios (ORs) and their corresponding 95% CI. Notably, HRs derived from multivariate analysis were applied in all analysis, except for specifically designated grouping analyses. Consistent with other meta-analysis, we also used chi-square test and *I*^2^ statistic to evaluate heterogeneity in STATA software with established codes. However, irrespective of the results of chi-square test and *I*^2^ statistics, the random models were used for all analyses for reducing the heterogeneity. We further explored the relationship between CD155 and the clinical characteristics and prognosis of cancer patients and the possible factors that contributed to heterogeneity through subgroup analyses. Moreover, the collected studies were grouped according to their publication date, detection method, sample size, analysis method, cutoff value, detected sample, and study region, and then meta-regression analysis was performed to find possible sources of heterogeneity. Sensitivity analysis was performed to evaluate the stability of this study. Begg’s and Egger’s tests were performed to analyze the publication bias.

## Results

### Literature search and study characteristics

**Fig. 1:**
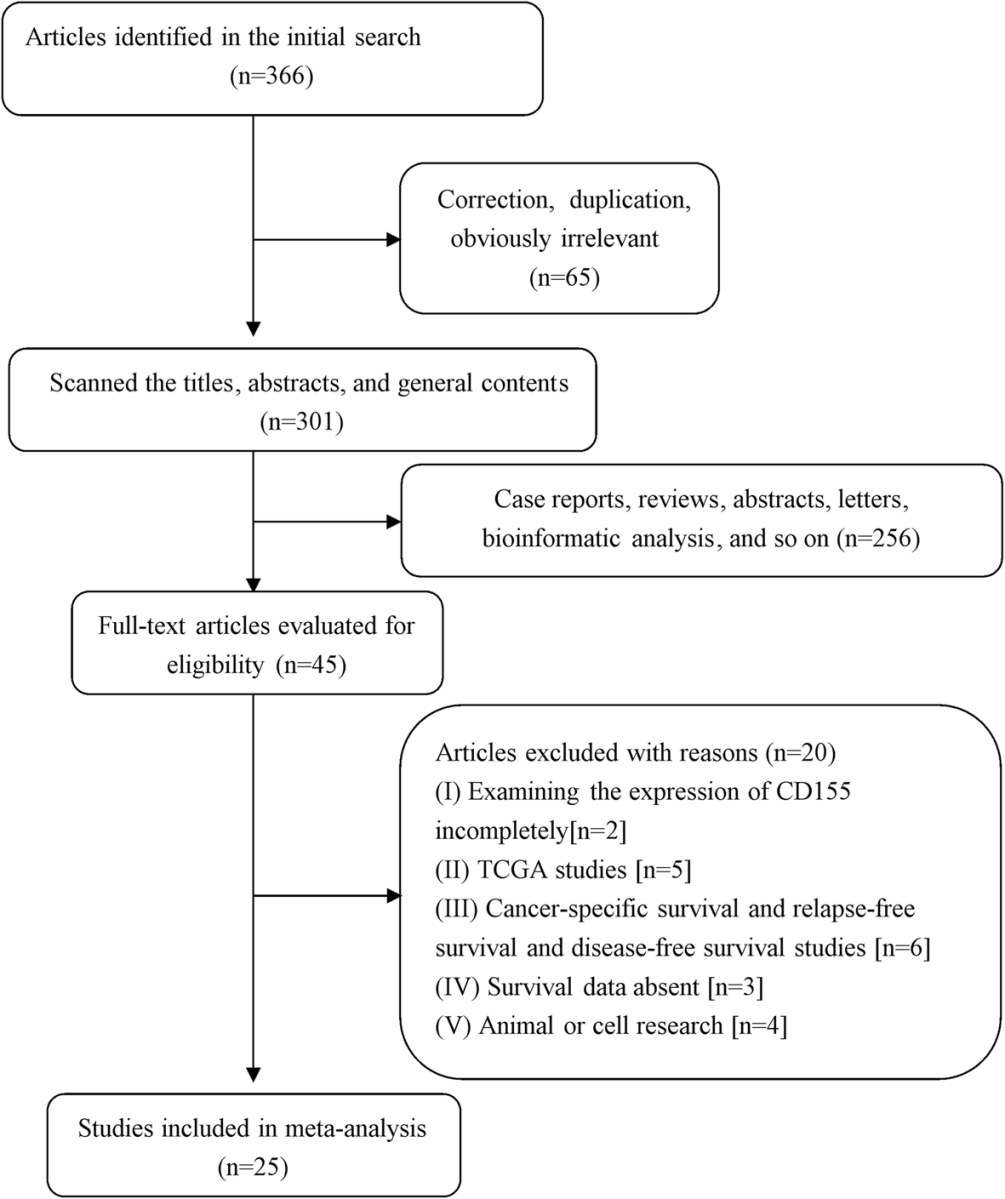
Flow chart of retrieving relevant articles from literature

The process followed to retrieve relevant articles from literature is shown in Fig. 1. Initial search yielded a total of 366 articles. These were further scanned for title, abstract, and general content, and 321 articles were excluded (correction, duplication, obviously irrelevant, etc. [*n* = 65]; case reports, reviews, abstracts, letters, bioinformatic analysis, and so on [*n* = 256]). Consequently, a total of 45 articles were selected for further screening. Among these, 20 were further excluded owing to different reasons. In particular, some of these studies involved incomplete examination of expression of CD155 (*n* = 2); TCGA studies (*n* = 5); cancer-specific survival, relapse-free survival, and disease-free survival studies (*n* = 6); the absence of survival data (*n* = 3); and animal or cell research (*n* = 4). Finally, the present study involved meta-analysis of 25 articles, published between 2013 and 2022. These articles contained 26 studies that involved 4325 cancer patients. The tumor types explored in these 26 studies included soft tissue sarcoma [26], hepatocellular carcinoma [27–30], acute myeloid leukemia [31], pancreatic cancer [32], cholangiocarcinoma [33], bladder cancer [34], lung cancer [35–38], esophageal cancer [39, 40], breast cancer [41–44], head and neck squamous cell carcinoma [45, 46], gastric cancer [47], gallbladder cancer [48], cervical adenocarcinoma [49], and colorectal cancer [50]. The study samples ranged from 43 to 444. Each study provided valid HRs based on reasonable cutoff values, obtained using univariate and (or) multivariate analysis. Most of the studies (22/26) were conducted in Asia, while the remaining ones were conducted in Europe. In particular, 23 studies examined CD155 expression in tumor tissues, wherein vast majority (20/26) of the studies examined CD155 expression by immunohistochemistry. The details of these studies are summarized in Table 1. Importantly, results based on the Newcastle-Ottawa quality assessment scale indicated that the included studies were of high quality (supplementary Table 1).

**Table 1:** Main characteristics of studies included in meta-analysis

Author (year)	Study region	Recruitment time	Follow-up time	Sample size	Cancer type	Detection method	Detected sample	Cutoff scores (high/low)	Analysis method	OS, HR estimation	Quality score
Atsumi S. (2013) [26]	Japan	NS	76.5 months (median)	43	Soft tissue sarcoma	qRT-PCR	Tissue	Optimal cutoff	Univariate analysis	0.94 (0.38–2.31)	7
Gong J. (2014) [27]	China	2008	2008–2012	96	Hepatocellular carcinoma	IHC	Tissue	Median	Univariate analysis	0.9383 (0.8824–0.9977)	8
Qu P. (2015) [28]	China	2009–2011	20 months (median)	174	Hepatocellular carcinoma	IHC	Tissue	> 0^a^	Univariate/multivariate analysis	0.518 (0.284–0.945)	8
Nishiwada S. (2015) [32]	Japan	1992–2010	Until 2014	134	Pancreatic cancer	IHC	Tissue	≥ ++^a^	Univariate/multivariate analysis	1.476 (1.019–2.139)	8
Huang D. W. (2017) [33]	China	2008–2015	19 months (median)	90	Cholangiocarcinoma	IHC	Tissue	> 3^a^	Univariate analysis	5.443 (2.822–10.498)	7
Stamm H. (2018) [31]	Germany/Australia	NS	NS	139	Acute myeloid leukemia	Flow cytometry	Cell lines	Optimal cutoff	Multivariate analysis	1.52 (1.04–2.23)	6
Stamm H. (2018) [31]	Germany/Australia	NS	NS	290	Acute myeloid leukemia	Flow cytometry	Cell lines	Optimal cutoff	Multivariate analysis	3.39 (1.45–7.94)	6
Zhang J. (2020) [34]	China	2008–2015	45 months (median)	228	Bladder cancer	IHC	Tissue	≥ 2^a^	Univariate/multivariate analysis	2.37 (1.6–3.5)	8
Xu Y. (2019) [35]	China	2008–2014	Until 2015	60	Lung cancer	IHC	Tissue	Median	Multivariate analysis	2.4 (1.05–5.5)	7
Sun H. (2019 [29])	China	2006–2010	Around 100 months	236	Hepatocellular carcinoma	Flow cytometry	Tissue	Optimal cutoff	Multivariate analysis	1.61 (1.00–2.61)	7
Yoshida J. (2019) [39]	Japan	2015–2017	NS	47	Esophageal cancer	ELISA	Serum	Optimal cutoff	Univariate/multivariate analysis	0.311 (0.068–1.086)	7
Yong H. (2019) [41]	China	2016–2018	NS	216	Breast cancer	IHC	Tissue	Optimal cutoff	Univariate/multivariate analysis	2.029 (1.059–3.887)	7
Stamm H. (2019) [42]	Germany	1991–2002	NS	197	Breast cancer	Flow cytometry	Tissue	Median	Multivariate analysis	1.822 (1.050–3.161)	7
Sun Y. (2020) [36]	China	NS	NS	334	Lung cancer	IHC	Tissue	> 0^a^	Univariate/multivariate analysis	1.372 (1.027–1.833)	6
Yao Y. (2020) [45]	China	2014–2015	NS	115	Head and neck squamous cell carcinoma	IHC	Tissue	Optimal cutoff	Univariate analysis	1.58 (1.09–2.3)	7
Li Y. C. (2020) [43]	China	2012–2013	75 months (median)	126	Breast cancer	IHC	Tissue	≥ ++^a^	Univariate/multivariate analysis	5.47 (1.42–20.99)	8
Wang J. B. (2020) [47]	China	2010–2014	More than 5 years	444	Gastric cancer	IHC	Tissue	≥ 4^a^	Univariate analysis	1.55 (1.19–2.01)	7
Albrecht T. (2021) [48]	Germany	1995–2016	NS	95	Gallbladder cancer	IHC	Tissue	Optimal cutoff	Univariate analysis	2.72 (1.35–5.47)	6
Murakami T. (2021) [49]	Japan	2004–2014	NS	67	Cervical adenocarcinoma	IHC	Tissue	≥ ++^a^	Univariate analysis	6.39 (2.04–19.98)	7
Yoshikawa K. (2021) [44]	Japan	2006–2018	NS	61	Breast cancer	IHC	Tissue	≥ 50^a^	Univariate analysis	2.99 (0.6–15.01)	6
Zhao K. (2021) [51]	China	2006–2018	Until 2019	114	Esophageal cancer	IHC	Tissue	≥ 2^a^	Univariate/multivariate analysis	1.646 (1.006–2.691)	7
Lee J. B. (2021) [37]	Korea	1998–2020	NS	259	Lung cancer	IHC	Tissue	Optimal cutoff	Univariate/multivariate analysis	1.36 (1.01–1.83)	6
Lim S. M. (2021) [46]	Korea	2005–2012	NS	375	Head and neck squamous cell carcinoma	IHC	Tissue	> 23^a^	Univariate analysis	1.4 (1.03–1.90)	6
Oyama R. (2022) [38]	Japan	2003–2006	NS	96	Lung cancer	IHC	Tissue	Optimal cutoff	Univariate analysis	3.74 (1.71–8.15)	8
Jin A. L. (2022) [30]	China	2012–2013	Until 2018	189	Hepatocellular carcinoma	IHC	Tissue	Optimal cutoff	Univariate/multivariate analysis	2.87 (1.51–5.45)	8
Murakami D. (2022) [50]	Japan	2013	More than 5 years	100	Colorectal cancer	IHC	Tissue	≥ ++^a^	Univariate analysis	2.17 (1.12–4.21)	7

^a^Scores were based on the multiplication or sum of intensity and distribution scores; *IHC* Immunohistochemical, *ELISA* Enzyme-linked immunosorbent assay,*NS* Data were not shown, *OS* Overall survival, *HR* Hazard ration

### Prognosis significance of CD155 expression in various cancer types

**Fig. 2:**
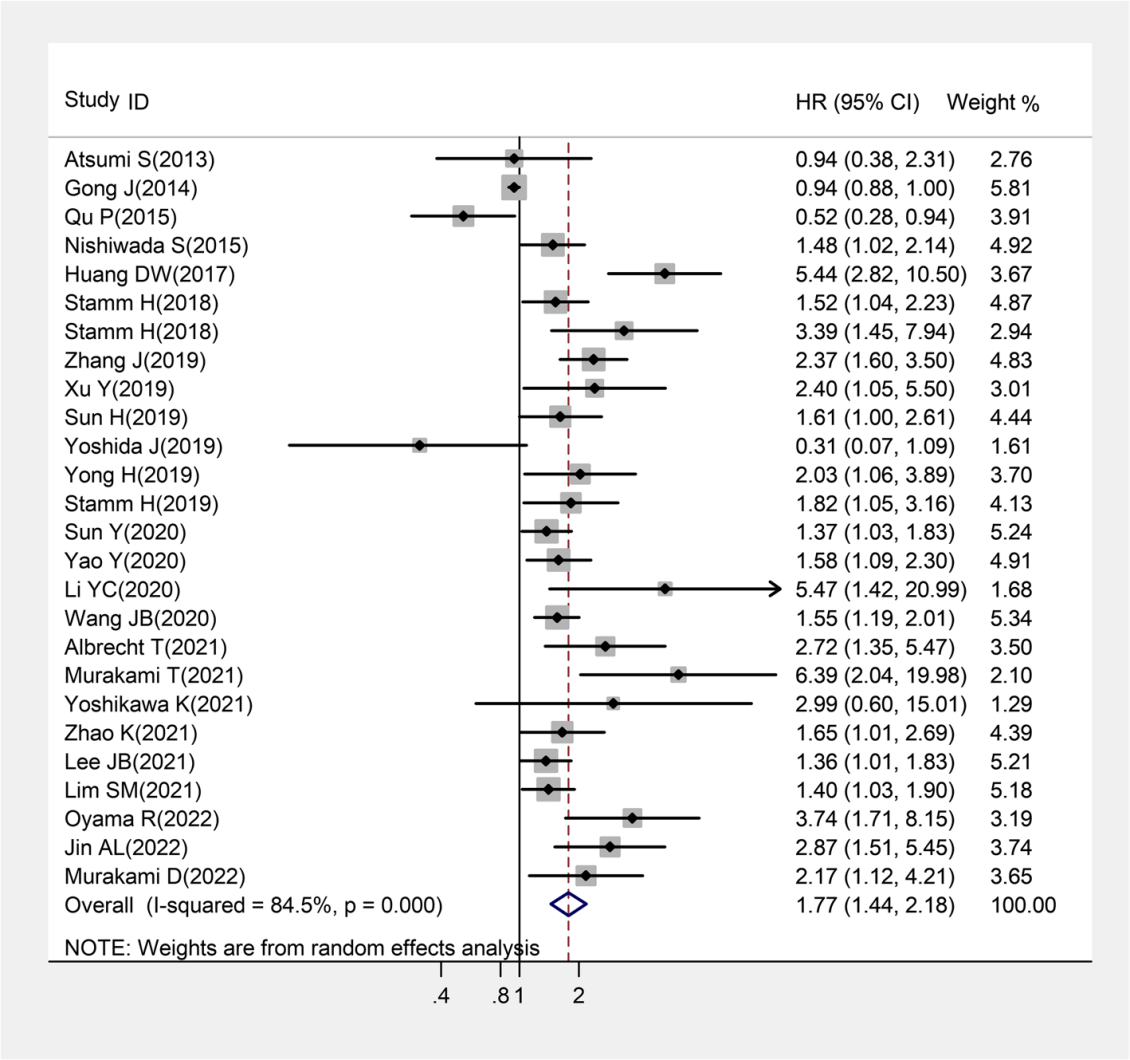
Forest plot for overall survival in patients with cancers by CD155 expression. (Increased CD155 expression was found to be significantly associated with reduced OS in patients with cancer as compared to low CD155 expression)

As shown in Table 2, increased CD155 expression was found to be significantly associated with reduced OS in patients with cancer as compared to low CD155 expression (pooled *HR* = 1.772, 95% *CI* = 1.441–2.178, *P* < 0.001, Fig. 2). In case of digestive system cancer, OS of the patients with high expression of CD155 was reported to be significantly lower as compared to the patients with low expression of CD155 (pooled *HR* = 1.570, 95% *CI* = 1.120–2.201, *P* = 0.009), and significant correlation was also obtained in hepatobiliary pancreatic cancer (pooled *HR* = 1.677, 95% *CI* = 1.037–2.712, *P* = 0.035) and digestive tract cancer (pooled *HR* = 1.512, 95% *CI* = 1.016–2.250, *P* = 0.042). Besides this, subgroup analysis demonstrated that the level of CD155 expression was significantly associated with OS in patients with breast cancer (pooled *HR* = 2.137, 95% *CI* = 1.448–3.154, *P* < 0.001), lung cancer (pooled *HR* = 1.706, 95% *CI* = 1.193–2.440, *P* = 0.003), and head and neck cancer (pooled *HR* = 1.470, 95% *CI* = 1.160–1.862, *P* = 0.001). Similar results were obtained for other cancer types, including soft tissue sarcomas, acute myeloid leukemia, bladder cancer, and cervical adenocarcinoma (pooled *HR* = 2.150, 95% *CI* = 1.348–3.428, *P* = 0.001). Significant differences in OS were also confirmed in multivariate analysis (pooled *HR* = 1.635, 95% *CI* = 1.319–2.027, *P* < 0.001) and univariate analysis (pooled *HR* = 1.792, 95% *CI* = 1.404–2.288, *P* < 0.001) groups. Importantly, it was observed that CD155 expression was associated with OS of cancer patients in the studies that were published < 3 years ago (pooled *HR* = 1.805, 95% *CI* = 1.493–2.182, *P* < 0.001) and at least 3 years ago (pooled *HR* = 1.568, 95% *CI* = 1.127–2.183, *P* = 0.008). Furthermore, a remarkable association was observed between OS and expression of CD155 in terms of different sample size (for sample size < 200, pooled *HR* = 1.839, 95% *CI* = 1.357–2.494, *P* < 0.001; for sample size > 200, pooled *HR* = 1.603, 95% *CI* = 1.365–1.881, *P* < 0.001), detection method used (for IHC, pooled *HR* = 1.863, 95% *CI* = 1.470–2.363, *P* < 0.001; for other methods, pooled *HR* = 1.519, 95% *CI* = 1.046–2.205, *P* = 0.028), and study region (for Asian region, pooled *HR* = 1.714, 95% *CI* = 1.369–2.146, *P* < 0.001; for Europe, pooled *HR* = 1.976, 95% *CI* = 1.411–2.769, *P* < 0.001).

**Table 2:** Meta-analysis of CD155 expression and prognosis in cancers

Categories	Studies (patients)	HR (95% *CI*)	*I*^2^ (%)	*P*_h_	*Z*	*P*
OS	26 (4325)	1.772 (1.441–2.178)	84.5	< 0.001	5.42	< 0.001
Cancer type						
Digestive system cancer	11 (1719)	1.570 (1.120–2.201)	87.9	< 0.001	2.62	0.009
Hepatobiliary pancreatic cancer	7 (1014)	1.677 (1.037–2.712)	90.0	< 0.001	2.11	0.035
Digestive tract cancer	4 (705)	1.512 (1.016–2.250)	51.7	0.054	2.04	0.042
Breast cancer	4 (600)	2.137 (1.448–3.154)	0.0	0.497	3.82	< 0.001
Lung cancer	4 (749)	1.706 (1.193–2.440)	58.8	0.063	2.93	0.003
Head and neck cancer	2 (490)	1.470 (1.160–1.862)	0.0	0.623	3.19	0.001
Others^a^	5 (767)	2.150 (1.348–3.428)	63.2	0.028	3.22	0.001
Analysis method						
Multivariate analysis	15 (2743)	1.635 (1.319–2.027)	61.3	0.001	4.48	< 0.001
Univariate analysis	21 (3403)	1.792 (1.404–2.288)	88.0	< 0.001	4.69	< 0.001
Publication date						
≥ 3 years	13 (1950)	1.568 (1.127–2.183)	86.6	< 0.001	2.67	0.008
< 3 years	13 (2375)	1.805 (1.493–2.182)	48.2	0.026	6.10	< 0.001
Size						
< 200	18 (1943)	1.839 (1.357–2.494)	85.3	< 0.001	3.92	< 0.001
> 200	8 (2382)	1.603 (1.365–1.881)	31.9	0.173	5.76	< 0.001
Detection method						
IHC	20 (3373)	1.863 (1.470–2.363)	86.7	< 0.001	5.14	< 0.001
Others	6 (952)	1.519 (1.046–2.205)	49.8	0.077	2.20	0.028
Study region						
Asian	22 (3604)	1.714 (1.369–2.146)	85.2	< 0.001	4.70	< 0.001
Europe	4 (721)	1.976 (1.411–2.769)	27.3	0.248	3.96	< 0.001

*OS* Overall survival, *HR* Hazard ration, *CI* Confidence interval, *P*_h_, *P*-value for heterogeneity based on *Q*-test; *P P*-value for statistical significance based on *Z*-test, *IHC* Immunohistochemistry; ^a^soft tissue sarcomas and acute myeloid leukemia and bladder cancer and cervical adenocarcinoma

### Correlation between CD155 expression and clinical characteristics of cancer

On the basis of included studies, the present study further collected valid clinical data to analyze the role of CD155 in cancer. As summarized in Table 3, a significant correlation was observed between CD155 expression and gender (pooled *OR* = 0.657, 95% *CI* = 0.510–0.846, *P* = 0.001), tumor stage (pooled *OR* = 1.697, 95% *CI* = 1.217–2.366, *P* = 0.002), LN metastasis (pooled *OR* = 1.953, 95% *CI* = 1.253–3.046, *P* = 0.003), and distant metastasis (pooled *OR* = 2.253, 95% *CI* = 1.235–4.110, *P* = 0.008), which indicated that high expression of CD155 was associated with advanced tumor stage and positive of LN metastasis and distant metastasis. However, no significant association was reported for age (pooled *OR* = 0.778, 95% *CI* = 0.512–1.181, *P* = 0.239), tumor size (pooled *OR* = 1.141, 95% *CI* = 0.610–2.136, *P* = 0.679), TNM stage (pooled *OR* = 1.829, 95% *CI* = 0.980–3.413, *P* = 0.058), or histologic grade (pooled *OR* = 1.793, 95% *CI* = 0.871–3.692, *P* = 0.113).

**Table 3:** Meta-analyses of CD155 expression classified by clinicopathological parameters

Study covariates	Studies (patients)	OR (95% *CI*)	*I*^2^ (%)	*P*_h_	*Z*	*P*
Gender (male/female)	10 (1472)	0.657 (0.510–0.846)	9.4	0.355	3.26	0.001
Age (< 60/≥ 60)	3 (366)	0.778 (0.512–1.181)	0.0	0.550	1.18	0.239
Tumor size (≤ 5/> 5 cm)	4 (622)	1.141 (0.610–2.136)	46.0	0.135	0.41	0.679
TNM stage (1–2/3–4)	6 (1062)	1.829 (0.980–3.413)	73.9	0.002	1.90	0.058
Tumor stage (T1 + T2/T3 + T4)	4 (810)	1.697 (1.217–2.366)	0.0	0.819	3.12	0.002
LN metastasis (absence/presence)	8 (1209)	1.953 (1.253–3.046)	62.1	0.010	2.95	0.003
Distant metastasis (absence/presence)	4 (625)	2.253 (1.235–4.110)	22.8	0.274	2.65	0.008
Histologic grade (well + moderately differentiated/poorly differentiated)	7 (1105)	1.793 (0.871–3.692)	75.3	< 0.001	1.58	0.113

*LN* Lymph node, *OR* Odds ratio, *CI* Confidence intervals, *P*_h_, *P*-value for heterogeneity based on *Q*-test; *P*, *P*-value for statistical significance based on *Z*-test

### Assessment of the heterogeneity, stability, and publication bias

**Fig. 3:**
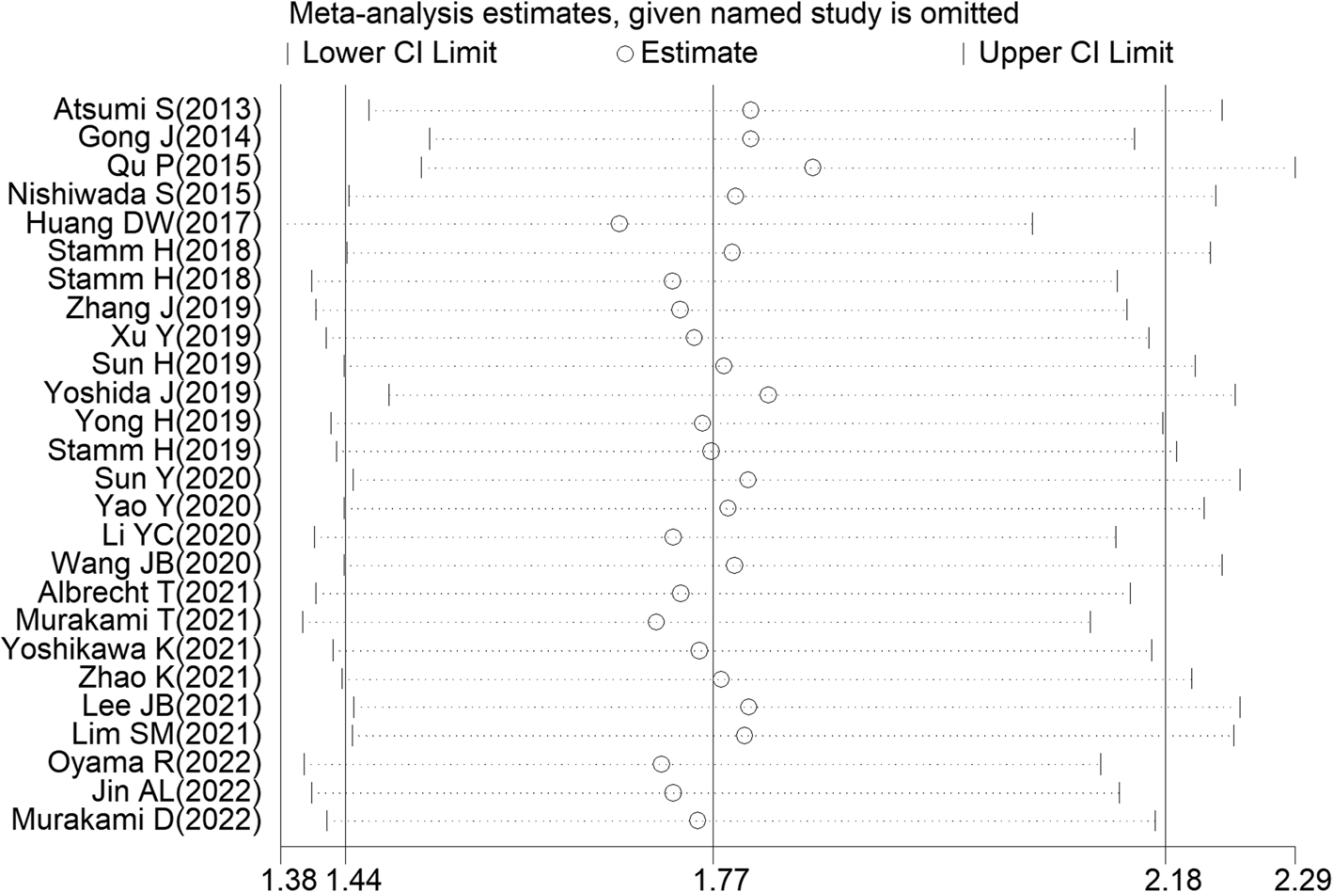
Sensitive analysis of overall survival for patients with cancers. (With the points estimated for the omitted individual dataset, all studies were distributed within 95% CI, which indicates that meta-analysis results were co-produced by each included study)

**Fig. 4:**
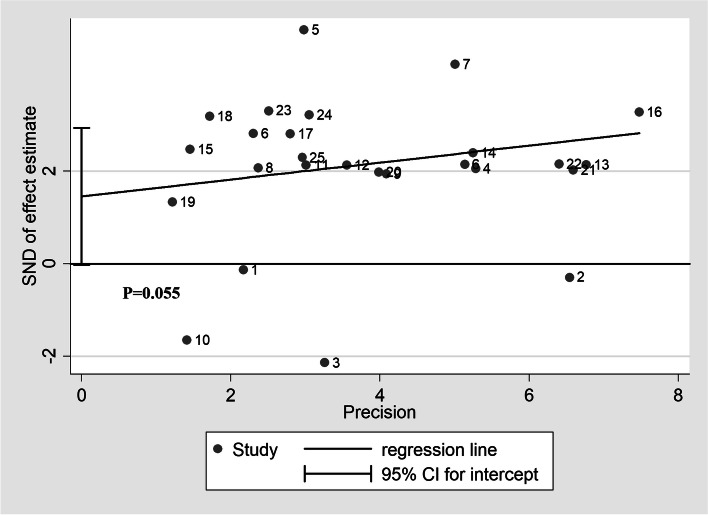
Assessment of publication bias of included studies in Egger’ test

To evaluate heterogeneity in this meta-analysis, chi-square test and *I*^2^ statistic were performed. As illustrated in Table 2, an extreme heterogeneity was observed in the assessment of overall HR for OS, by employing a random-effects model (*I*^2^ = 84.5%, *P*_h_ < 0.001). However, further subgroup analysis revealed that most of the heterogeneity was still remarkable. Therefore, for consistency and to reduce heterogeneity, random-effects models were employed for all analyses. The study also conducted meta-regression analysis to find possible source of heterogeneity. Unfortunately, publication date (*P* = 0.571), detection method (*P* = 0.542), sample size (*P* = 0.990), analysis method (*P* = 0.684), cutoff value (*P* = 0.847), detected sample (*P* = 0.707), and study region (*P* = 0.205) were not identified as the main sources of heterogeneity. Besides this, sensitivity analysis was applied to evaluate individual impact of each study. As shown in Fig. 3, with the points estimated for the omitted individual dataset, all studies were distributed within 95% CI, which indicates that meta-analysis results were co-produced by each included study. Moreover, given the negative results of Egger’s (*P* = 0.055, Fig. 4) and Begg’s tests (*P* = 0.158), it was concluded that there was no publication bias in the meta-analysis. Altogether, it was believed that conclusions of this meta-analysis were robust and credible.

## Discussion

In the recent years, there has been significant increase in cancer incidence and mortality rate worldwide [4]. Importantly, cancer not only affects the patient’s health but it also harms their families as well. Thus, it is important to devise strategies to improve quality of life and extend life expectancy of the patients. To alleviate this situation, various strategies, including early detection methods, innovative surgical techniques, checkpoint blockade immunotherapies, and targeted therapies, have been applied in the recent years. Encouragingly, previous studies identified a series of profound molecules that played a significant role in cancer development. Besides this, these factors were also associated with tumor size, tumor grade, and lymph node metastasis. Importantly, these molecules more or less showed abnormal expression and suspicious biological activity in various cancer types. In fact, the tumor process could be inhibited or even stopped by interfering or blocking these molecules [2, 12, 23, 37]. Molecular biomarkers, such as RNA, lncRNA, and proteins, are used with clinical information to enable experimental to clinical translation, thereby improving patient care and outcomes [51–53]. These successful discoveries have assisted in reversing the current crisis observed in the field of oncology, with remarkable speed. However, significant differences have been reported in various research results. In addition to this, uncertainties in clinical application of these strategies further limit their use. Therefore, it is necessary to explore more suitable interventions that would be more conducive in clinical practice and would provide new direction for medical and health work.

CD155 is an adhesion molecule that was first discovered in a study focused on poliovirus infection [54]. The mechanism of CD155 in cancer has been extensively studied in the past. CD155 is now considered as a member of immunoglobulin superfamily, which is characterized by four splice isomers. In particular, *α*-isomers contain immunoreceptor tyrosine-based inhibitory motif (ITIM) that is known to be essential for intrinsic biology of tumor cells [5, 6]. CD155 is expressed in humans as a membrane-bound protein encoded by CD155α and CD155δ and as a soluble protein encoded by CD155β and CD155γ that lacks transmembrane regions. At present, the physiological effects of CD155 mainly depend on αvβ3 integrin. However, the biological functions of the *β* and γ isoforms, which lack transmembrane domains, remain unclear [9]. Previous studies reported that up-regulated expression of CD155 in numerous malignant tumors. In particular, abnormal expression of CD155 might be dependent on the activation status of DNA damage-response pathway, Raf/MEK/ERK/activator protein-1 signaling pathway, and sonic hedgehog signaling pathway. It has been previously reported that toll-like receptor agonists could enhance the expression of CD155 in tumor immune cells [10, 22, 55]. Evidence provided by previous studies suggested that CD155 significantly correlated with unfavorable clinicopathological features and prognosis of certain cancer types. Importantly, CD155 was reported to play a crucial role in adhesion, migration, differentiation, proliferation, survival, and metastasis of tumor cells [31–34]. Similarly, the absence of CD155 exerted an inhibitory effect on tumor growth and metastasis, and blocking of PD-1 or PD-1 and CTLA4 was found to be more effective in the absence of CD155 [56]. In addition to this, TIGIT is expressed on T and NK cells. It encoded immunoglobulin domains and an ITIM and exhibited up-regulated expression in cancer, where it was associated with poor clinical prognosis [6]. And TIGIT could bind to CD155, CD112, and CD113, which are ligands on tumor cells. Among them, CD155 has been shown to be superior to other ligands for TIGIT with high affinity [17–19]. When CD155 is up-regulated in tumor cells, co-stimulatory molecule DNAM-1 recognizes and binds to tumor cells to stimulate immunity, while the inhibitory receptor TIGIT induces intracellular signaling to exert inhibitory effects. Therefore, DNAM-1 and TIGIT compete to bind CD155 to produce different results; however, emerging evidence has found that CD155 has the highest binding ability to TIGIT [57]. Importantly, TIGIT is considered a promising target for its immunomodulatory role in carcinogenesis; at the same time, CD155/TIGIT, a novel immune checkpoint in human cancers, can exert inhibitory effect on PI3K/MAPK signaling, NF-κB signaling, and AKT/mTOR signaling, which lead to downregulate metabolism, suppress cytokine production, and inhibited NK cell cytotoxicity [15, 58]. However, owing to the limitation of current clinical and technical means, related studies could not be unified. Several studies have previously suggested that CD155 played a dual role in various cancer types. Atsumi et al. showed that there was no significant difference in OS among patients with different CD155 levels [26]. Similarly, high CD155 expression did not increase tumor burden [39]. Additional evidence demonstrated that CD155 expression was decreased in tumor cell, and loss of CD155 expression in cancer patients was associated with worse prognosis [28]. In fact, CD155 played different roles in different stages of tumors. It has also been reported that CD155 expression in different cell structures exerted obvious differences in immunoregulatory function and clinical impact [5, 16]. In the present study, a scientific meta-analysis was conducted on existing literature, to explore the clinical value of CD155 in various cancer types, and thus provide guidance for subsequent studies.

Current meta-analysis concluded that CD155 acted as an independent marker of prognosis in cancer patients. The study involved 26 studies, which included 4325 cancer patients, and was first to reveal an association between increased CD155 expression and reduced OS in cancer patients by meta-analysis. Interestingly, the statistical significance in the subgroup analysis of hepatobiliary pancreatic cancer and digestive tract cancer was affirmed; meanwhile, a strong association was identified in digestive system cancer, breast cancer, lung cancer, head and neck cancer, and other cancer types included in this analysis, which indicated that CD155 might play a consistent and compatible role in various cancers. In addition to this, no variations were recorded in prognostic value of CD155 in terms of analysis method, publication date, sample size, detection method, and study region, which could be easily observed in the subgroup analysis. These results also supported the stable role of CD155 in various cancers. Furthermore, significant heterogeneity was observed in the present analysis without suspense. Despite this kind of situation, the exploration and analysis were continued. So far, no clarity is available on the same. Importantly, publication date, detection method, sample size, analysis method, cutoff value, detected sample, and study region did not serve as the main source of heterogeneity. Therefore, random-effects models were used throughout the study to mitigate some heterogeneity. Moreover, the study further analyzed the relationship between CD155 and clinical characteristics, and it was observed that abnormal expression of CD155 was related to tumor stage, LN metastasis, and distant metastasis, which meant that differential expression of CD155 in various cancers might lead to different biological responses in the patients. In particular, high expression of CD155 would make cancer more aggressive and lead to advanced tumor stage and positive of LN metastasis and distant metastasis. It is known that significant differences exist in the mechanisms of CD155 in cancer progression; however, clinical evidence collected and analyzed in this meta-analysis strongly suggested that CD155 played an effective role in cancer development, and CD155 might ultimately act as a pro-oncogenic factor in tumorigenesis.

Importantly, conclusions drawn regarding the correlation between CD155 and cancer need to be drawn prudentially, primarily owing to the limitations of this meta-analysis. Besides this, there are certain issues that need to be considered. In particular, the present study included only a few articles and many types of cancer, so the data was obviously insufficient to analyze the prognosis and clinical characteristics of a single type of cancer. Despite all the efforts, the HRs for some of the studies were calculated from survival curve, and the detection methods and cutoff values for CD155 expression were not uniform, which was bound to have certain statistical errors. The study mainly involved studies from Asia and Europe, and there was lack of information on other continents and races. The study involved inevitable heterogeneity. Lastly, this study presented only a comprehensive analysis of a single molecule of CD155. Sufficient data could not be obtained for combined analysis of its related molecules. Thus, future studies must involve different geographical regions and populations. Additionally, future studies must be carried out in more number of patients, and more rigorous experiments should be included, to better understand the role of CD155 in cancer.

## Conclusion

Altogether, the presented meta-analysis confirmed that over-expression of CD155 was associated with advanced tumor stage, positive of LN metastasis and distant metastasis, and worse OS. Therefore, it could be concluded that CD155 played a crucial role in cancer, and it could provide a strong/new direction for exploring/devising new strategies for cancer diagnosis and treatment.

## Supplementary Information

**Additional file 1.** PRISMA NMA Checklist.

**Additional file 2.** Supplementary search strategies.

**Additional file 3: Supplementary Table 1.** The Newcastle-Ottawa Quality Assessment Scale of studies included in meta-analysis.

## Data Availability

All data are available from the corresponding author.

## Abbreviations

ORs: Odds ratios
HRs: Hazard ratios
CIs: Confidence intervals
OS: Overall survival
LN: Lymph node
