# Supplementary material for: Prognostic and clinicopathological significance of CD155 expression in cancer patients: a meta-analysis
Source: World J Surg Oncol. 2022 Oct 29;20:351. doi: 10.1186/s12957-022-02813-w (PMC9617385; doi:10.1186/s12957-022-02813-w)
Supplement: Supplementary file 2 — Additional file 2. Supplementary search strategies. [file 12957_2022_2813_MOESM2_ESM.docx]

Search strategies:

We conducted systematic retrieval through PUBMED as follow: ("CD155") AND ("cancer" OR "tumor" OR "neoplasm" OR "carcinoma") AND ("prognosis" OR "Prognostic" OR "survival" OR "outcome")

URL:https://pubmed.ncbi.nlm.nih.gov/?term=%28%22CD155%22%29+AND+%28%22cancer%22+OR+%22tumor%22+OR+%22neoplasm%22+OR+%22carcinoma%22%29+AND+%28%22prognosis%22+OR+%22Prognostic%22+OR+%22survival%22+OR+%22outcome%22%29&sort=date.

We conducted systematic retrieval through WEB OF SCIENCE as follow: ("CD155") AND ("cancer" OR "tumor" OR "neoplasm" OR "carcinoma") AND ("prognosis" OR "Prognostic" OR "survival" OR "outcome")

URL:https://www.webofscience.com/wos/woscc/summary/d603289f-4d3b-4126-af21-507670ba52fa-55160b5d/relevance/1

PMC and other web databases were further used to search the full text and related references. Meanwhile, other aliases of CD155 such as PVR, NECL-5, and TAGE-4 were also substituted in the retrieval formula and retrieved them one-by-one.
