# Supplementary material for: Prognostic and clinicopathological significance of CD155 expression in cancer patients: a meta-analysis
Source: World J Surg Oncol. 2022 Oct 29;20:351. doi: 10.1186/s12957-022-02813-w (PMC9617385; doi:10.1186/s12957-022-02813-w)
Supplement: Supplementary file 3 — Additional file 3: Supplementary Table 1. The Newcastle-Ottawa Quality Assessment Scale of studies included in meta-analysis. [file 12957_2022_2813_MOESM3_ESM.docx]

Supplementary table 1. The Newcastle-Ottawa Quality Assessment Scale of studies included in meta-analysis

| Author(Year) | Selection | Comparability | Exposure | Quality score |
| --- | --- | --- | --- | --- |
| Atsumi S(2013) | 3 | 2 | 2 | 7 |
| Gong J(2014) | 4 | 2 | 2 | 8 |
| Qu P(2015) | 4 | 2 | 2 | 8 |
| Nishiwada S(2015) | 4 | 2 | 2 | 8 |
| Huang DW(2017) | 3 | 2 | 2 | 7 |
| Stamm H(2018) | 2 | 2 | 2 | 6 |
| Stamm H(2018) | 2 | 2 | 2 | 6 |
| Zhang J(2019) | 3 | 2 | 3 | 8 |
| Xu Y(2019) | 3 | 2 | 2 | 7 |
| Sun H(2019) | 3 | 1 | 3 | 7 |
| Yoshida J(2019) | 3 | 2 | 2 | 7 |
| Yong H(2019) | 3 | 2 | 2 | 7 |
| Stamm H(2019) | 3 | 2 | 2 | 7 |
| Sun Y(2020) | 2 | 1 | 3 | 6 |
| Yao Y(2020) | 3 | 1 | 3 | 7 |
| Li YC(2020) | 3 | 2 | 3 | 8 |
| Wang JB(2020) | 4 | 2 | 1 | 7 |
| Albrecht T(2021) | 3 | 1 | 2 | 6 |
| Murakami T(2021) | 3 | 2 | 2 | 7 |
| Yoshikawa K(2021) | 3 | 1 | 2 | 6 |
| Zhao K(2021) | 3 | 2 | 2 | 7 |
| Lee JB(2021) | 2 | 2 | 2 | 6 |
| Lim SM(2021) | 2 | 2 | 2 | 6 |
| Oyama R(2022) | 3 | 2 | 3 | 8 |
| Jin AL(2022) | 4 | 2 | 2 | 8 |
| Murakami D(2022) | 3 | 2 | 2 | 7 |
